# Biomaterials Tailoring at the Nanoscale for Tissue Engineering and Advanced Therapies

**DOI:** 10.3390/nano11051221

**Published:** 2021-05-06

**Authors:** Monica Boffito, Gianluca Ciardelli

**Affiliations:** Politecnico di Torino, Department of Mechanical and Aerospace Engineering, Corso Duca degli Abruzzi 24, 10129 Turin, Italy

The definition of the term “biomaterial” dates back to 1991, during the 2nd Consensus Conference on the Definitions in Biomaterials organized by the European Society of Biomaterials in Chester (UK). In detail, as an outcome of the Consensus Conference a biomaterial has been defined as “a material intended to interface with biological systems to evaluate, treat, augment or replace any tissue, organ or function of the body” [1]. Although the plethora of available biomaterials for biomedical applications is continuously evolving, including an increasing number of custom-made, newly developed and multi-component biomaterials, the validity of this definition is still undeniable. However, the growing knowledge on the interfacial interactions occurring between biomaterials and biological systems and the continuous advancements in biomaterial synthesis and functionalization procedures, have made biomaterial development and processing one of the key challenges of current basic and advanced research in the biomedical field. As a matter of fact, the PubMed database clearly highlights that the number of publications related to the “biomaterial” field has exponentially increased over the last decades, with a maximum of 15,804 published works in 2019.

The interaction of biomaterials with biological systems occurs at different length-scales, namely at the macro-, micro- and nano-scales. In particular, the design of materials and structures with nanometric precision has demonstrated its enormous potential in controlling cellular processes such as adhesion, proliferation and differentiation [2]. On the other hand, nanosized carriers have shown enhanced efficacy and precision in delivering therapeutic or diagnostic payloads to specific living environments, thus representing powerful tools in both the diagnosis and treatment of pathological states [3,4].

In this scenario, the Special Issue “Biomaterials Tailoring at the Nanoscale for Tissue Engineering and Advanced Therapies” offers to the readers a series of articles describing breakthrough research in the field of bionanotechnology and its exploitation in tissue engineering/regenerative medicine as well as in drug delivery and cell therapy. The issue is comprised of 11 peer-reviewed manuscripts (three Reviews and eight Original Research Articles) focused on different fields addressing the selected topic, namely drug carrier design and consequent payload delivery, surface property tuning at the nanoscale through functionalization and fabrication procedures, and stimuli-sensitive nanoparticle design and application. The development of nano-features thus represents the narrative thread of the whole manuscripts comprised in the Special Issue. Interestingly, the 80 contributors, scientists from universities and research institutes from nine countries (i.e., Czech Republic, Greece, Iraq, Italy, Korea, Portugal, Singapore, Spain and United Kingdom), described, collectively, biomaterials with nano-scale features exploited in different aspects of the whole medical device development pipeline. For instance, Fiorilli et al. developed smart nano-biomaterials by providing mesoporous bioactive glasses with chemical and biological cues exerting promising therapeutic effects (i.e., pro-osteogenic, anti-clastogenic) for the management of patients with compromised bone remodeling, such as those affected by osteoporosis fractures [5]. In detail, the authors developed strontium-doped mesoporous bioactive glasses surface functionalized with a soluble recombinant form of ICOS (ICOS-Fc) through carbodiimide chemistry. The proposed approach combined the well-known excellent bioactivity of bioactive glasses with the release of pro-osteogenic strontium ions and the inhibition of osteoclast activity provided by the surface grafted ICOS-Fc. A different functionalization process was exploited by Choi and co-workers to surface modify nanodiamonds with icariin (ICA) [6]. In detail, the authors first surface modified nanodiamonds with dopamine hydrochloride (DOPA) and then functionalized the modified nano-biomaterials with ICA though DOPA-anchoring. As a result, they achieved a prolonged release of ICA up to 4 weeks, which significantly enhanced the osteogenic differentiation capacity of nanodiamonds as such. In the field of tissue engineering, hydroxyapatite (HA) is a nano-biomaterial widely investigated for its bioactivity and biomimesis of the native inorganic phase of bone. However, polymers with low melting temperature (<200 °C), such as poly(methyl methacrylate), poly(lactic-*co*-glycolic acid), or polyethylene, which are widely used in bone, dental and maxillofacial engineering, are bioinert in their native form and, importantly, unsuitable for traditional procedures applied for HA coating deposition (i.e., thermal spray, vapor phase coating, plasma spray). Taking into account this important issue, Riau and colleagues surveyed in their review the available approaches for HA deposition on the surface of low melting temperature polymers (i.e., the biomimetic deposition and direct nanoparticle immobilization techniques), focusing on their key features, pros and cons, with the final aim of providing readers with an in-depth overview useful in decision making processes during bone tissue engineering device development [7]. Another class of biomaterials with a huge promise in the biomedical field includes melanin and melanin-like materials. These polymers have been widely investigated as coatings for tissue engineering substrates, promoting the adhesion and proliferation of different cell phenotypes as well as the healing cascade of skin, bone, and nerve defects in vivo. In this regard, Cavallini et al. provided readers with an up-to-date survey of recent advances and still open challenges in melanin-based materials design and application [8]. The authors initially focused on melanin potential in the biomedical field and the available strategies for melanin-like material synthesis; then, they provided an overview on hybrid systems resulting from the combination of melanin or melanin-like materials with inorganic and/or organic components. Finally, they reported on the application of melanin and melanin-like materials in the regenerative medicine field, with particular attention to wound healing and bone, cartilage, nerve and muscular tissue engineering. Interestingly, in the field of neural and muscular tissue engineering, melanin and melanin-like polymers hold great promise due to their conductive properties and self-healing ability. Physico-mechanical stimulation is another emerging approach to regulate cell behavior and differentiation potential. In this regard, Losi et al. reported on the design of a bioactive nanostructured topical medication promoting dermal tissue regeneration, comprising a bilayered fibrin/poly(ether urethane) membrane obtained through a combination of electrospinning and spray phase inversion methods [9]. Additional loading of platelet lysate into the fibrin counterpart of the device significantly accelerated wound healing in diabetic mice and favored both re-epithelialization and collagen deposition. Another strategy to physically stimulate biological processes encompasses the use of magnetic nanoparticles. Song and colleagues combined a magnetic torque stimulation system with lectin surface functionalized magnetic particles, resulting in the possibility to apply forces on the surface of cells operating in a noncontact mode [10]. The developed systems turned out to be able to stimulate both cardiomyocytes and cardiac fibroblasts and induce the upregulation of hypertrophic markers in hypoxic cardiomyocytes. These preliminary results could have relevant implications in the development of in vitro models of the cardiac tissue suffering from mechanically associated diseases, such as hypertrophy. In another work, Carvalho and colleagues combined a methacrylated gellan gum hydrogel with magnetoelectric microspheres based on poly(L-lactic acid) and magnetostrictive cobalt ferrites to develop a new matrix for bone tissue engineering [11]. The designed hybrid matrices effectively mimicked the natural electromechanical environments of the human body, resulting in enhanced proliferation rate of preosteoblast cells under mechano-electrical stimulation provided by the hydrogel and the embedded magnetoelectric particles. Embedding nanoparticles into a hydrogel phase is a promising strategy to vehicle a therapy in the target area of the body, while allowing its prolonged and sustained release *in loco*. A notable example in this field is provided in the work by Boffito, Laurano et al. that embedded ibuprofen-loaded mesoporous carbons into an injectable thermosensitive hydrogel based on a customized Poloxamer 407-based poly(ether urethane) [12]. Particles embedded into the thermosensitive hydrogels did not negatively influence their thermoresponsiveness and temperature-induced gelation potential. Moreover, the developed hybrid formulations successfully released ibuprofen over time, thus opening up towards the development of easily injectable formulations able to locally and progressively deliver their cargo, thus enabling the treatment of many pathological states (e.g., as topical medications to treat chronic wounds). In the field of infected wound healing, Dorazilová et al. developed collagenous scaffolds showing antibacterial properties provided by the synergistic effect of chitosan and selenium nanoparticles [13]. Even at low concentration, selenium nanoparticles embedded into the porous constructs induced a strong antibacterial effect against Gram-positive bacterial strains and methicillin-resistant *Staphylococcus aureus* and *Staphylococcus epidermidis*. Finally, in the wide plethora of nanomaterials investigated for biomedical applications, those of natural origin or based on naturally derived building blocks (e.g., amino acids) hold a huge promise due to their biomimetic and bioactive properties and their excellent biological activity. For instance, extracellular vesicles (EVs) are nanoscale heterogeneous cell-secreted membrane vesicles, with a tremendous potential in the biomedical field thanks to their no tumorigenicity, low immunogenic potential and high stability. In this regard, Saludas and colleagues provided a thorough overview on the application of EVs in the treatment of cardiac damage after myocardial infarction [14]. Peptide-based nanocarriers are another notable example of nanomaterials based on biological building blocks, namely amino acids linked by peptide bonds. Such nanocarriers exhibit various advantages, including their low cytotoxicity, high drug-loading capability and high compositional versatility. Al-azzawi et al. exploited this last feature to ad hoc synthesize a peptide delivery system presenting an ApoE-mimicking peptide and two flurbiprofen molecules for the treatment of Alzheimer’s disease [15]. The ApoE-mimicking peptide enhanced the transport of the nanocarrier through an in vitro model of the blood-brain barrier via receptor-mediated transcytosis, while flurbiprofen was released in its active form though hydrolysis in a lysosome-mimicking medium. The proposed approach could represent a significant advancement towards the development of efficient drug delivery systems able to cross the blood-brain barrier and release their payload in the brain tissue.

In summary, this Special Issue reflects the high diversity, versatility and existing creativity in the design of nano-biomaterials and their application in the biomedical field. Although this paper collection cannot be completely exhaustive, the reader will find a comprehensive overview of the latest advancements in biomaterials tailoring at the nanoscale for advanced applications in tissue engineering and drug delivery. Moreover, the reader will easily appreciate that the addressed topic is broad in nature and represents a still open question in the biomedical field. The available tools for the development of nano-biomaterials are continuously expanding and the strict collaboration among researchers with different know-how (e.g., biochemistry, materials science, biomedical engineering, biology) will allow in the near future to further advance the fascinating and supradisciplinary field of bionanotechnology, finally leading to the translation of basic research results from the bench to the bedside.
